# The association of COVID-19 with the development of acute avascular necrosis of the head of the femur, apart from steroid usage

**DOI:** 10.5339/qmj.2025.38

**Published:** 2025-06-18

**Authors:** Saurabh Sharma, Akhil Bhansal, Rehan Khan, Suresh Uikey, Jonsi Tavethia, Anshul Bhadania, Shubh Mehta, Kinjal Solanki, Prahar Darji, Kavya Darji, Kamal Sharma

**Affiliations:** 1 Department of Orthopedics, Gandhi Medical College, Bhopal, India; 2 Department of Internal Medicine, B.J Medical College, Ahmedabad, India; 3 B.J. Medical College, Ahmedabad, India; 4 GCS Medical College & Research Center, Ahmedabad, India; 5 U N Mehta Institute of Cardiology and Research Centre, Ahmedabad, India *Email: anshulbhadania@gmail.com

**Keywords:** Avascular necrosis, COVID-19, steroid use, risk factors, retrospective study

## Abstract

**Background:** The SARS-CoV-2 pandemic (COVID-19) has significantly impacted global health, with emerging evidence indicating potential long-term complications affecting various organ systems, including the musculoskeletal system, like avascular necrosis (AVN) of the femoral head. This retrospective study aims to investigate the incidence and risk factors of AVN in patients treated for COVID-19.

**Methods:** We conducted a cross-sectional retrospective study in the department of orthopedics at a tertiary care teaching hospital in Central India from July 2022 to December 2023. Patients presenting with new-onset hip pain and low back pain who were asymptomatic before COVID-19 with a new radiological diagnosis of AVN hip were included in the study. Data on demographics, comorbidities, steroid use, and COVID-19 management were collected and analyzed using statistical tests to identify associations between these factors and AVN incidence.

**Results:** A total of 86 patients met the inclusion criteria. The majority of participants were males (83.7%), predominantly within the 30–45 years (44.2%) and 15–30 years (30.2%) age groups. Bilateral AVN was observed in 62.8% of cases. A significant portion (25.6%) had a history of COVID-19, with steroid use prevalent among 30.2% of participants, with an odds ratio of 4.47 indicating strong association. Statistically significant associations were found between COVID-19 status and age distribution (*p* = 0.049), comorbidities (*p* = 0.014), symptom onset (p = 0.001), and steroid therapy history (*p* = 0.002).

**Conclusions:** This study highlights a notable incidence of AVN among COVID-19 patients, with significant correlations to steroid use and specific comorbidities. The findings underscore the importance of vigilant monitoring for AVN in post-COVID-19 patients, particularly those with a history of steroid therapy. Further research is needed to elucidate the mechanisms linking COVID-19 and AVN and to develop targeted prevention strategies.

## INTRODUCTION

Osteonecrosis, also known as avascular necrosis (AVN), is a degenerative bone condition that results from the death of bone cells due to disrupted blood supply to the subchondral bone, predominantly of the epiphyses of weight-bearing long bones, such as the femoral head, knee, talus, and humeral head, with the hip being the most commonly affected joint.^1^ Reduced blood flow to the bone can occur through three primary mechanisms: intravascular occlusion from thrombi or fat emboli, intraosseous extravascular compression due to lipocyte hypertrophy or Gaucher cells, and vascular interruption resulting from fractures or dislocations.^2^

The prevalence of AVN varies globally, with estimates indicating 15,000–20,000 new cases annually in the United States alone.^3^ Beyond its initial respiratory effects, the SARS-CoV-2 pandemic has impacted multiple organ systems, leading to significant long-term complications. AVN of the femoral head, in particular, has gained attention due to its severe impact on mobility and quality of life. Emerging evidence suggests that COVID-19 may exacerbate or even directly cause AVN.^4^ It has been demonstrated that COVID-19 infection causes a systemic inflammatory response that profoundly affects a number of organs and tissues, including the bones. Systemic inflammation triggered by the virus can negatively affect bone and joint health by elevating cytokines such as CXCL10, interleukin-17 (IL-17), and tumor necrosis factor-alpha (TNF-α), which impair osteoblast function and bone regeneration.^5^ Pro-inflammatory cytokines like IL-6 and TNF-α, which are integral to the pathophysiology of COVID-19, are overproduced during this inflammatory response. Increased resorption of bone and impaired creation of new bone are linked to elevated levels of these cytokines, which can result in problems such as osteoporosis and AVN of the bone.^6–8^

Furthermore, increased vascular permeability brought on by the COVID-19-related inflammatory response could trigger edema and raised bone pressure. This disorder can boost the risk of ischemia and consequent bone necrosis by impairing blood flow and oxygen delivery to the bone tissue.^6,9^ In addition to its effects on the lungs, systemic inflammation also has a significant impact on the vascular system, leading to thromboembolic events that can worsen the blood supply to the bones.^9^ COVID-19 itself is associated with coagulopathies that induce a hypercoagulable state, potentially causing blood vessel obstruction and compromised blood supply due to stasis.^10^

The interplay between inflammation and coagulation in COVID-19 patients highlights the complexity of the disease and the need for comprehensive management strategies that address both inflammatory and thrombotic complications.

Additionally, corticosteroids, widely used to manage severe COVID-19 infections for their anti-inflammatory effects, can lead to complications, including acute respiratory distress syndrome, disseminated intravascular coagulation, and multiple organ failure. Despite their benefits, corticosteroids are also linked to potential adverse effects, including AVN.^11^ Depending on the dosage, length of treatment, and specific patient risk factors, the incidence of AVN in patients using corticosteroids can vary greatly, with estimates ranging from 3% to 40%.^12,13^

Osteocytes and osteoblasts, which are essential for bone health and regeneration, have been shown to undergo apoptosis in response to corticosteroids, such as dexamethasone and methylprednisolone^14,15^ The apoptotic process is frequently facilitated by many signaling pathways, such as the PI3K/Akt and Wnt/β-catenin pathways, which are essential for preserving bone homeostasis.^14,16^ Overuse of glucocorticoids might worsen inflammation and oxidative stress, which exacerbates bone necrosis.^17,18^ Moreover, glucocorticoids have the ability to cause hypercoagulability, which exacerbates ischemia and vascular impairment in the femoral head.^19^

This study aims to investigate the incidence and risk factors associated with AVN in patients treated for COVID-19, with a particular focus on the potential links between steroid use and specific comorbidities.

## METHODOLOGY

### Study design

This study was conducted as a cross-sectional retrospective analysis. The study included patients who visited an orthopedics outpatient department between the period of July 2022 to December 2023. Patients who met the specified inclusion and exclusion criteria were considered for enrollment.

### Study population

A total of 86 patients who met all the inclusion criteria and none of the exclusion criteria were enrolled for the study.

**Inclusion Criteria**—Patients were included in the study if they presented with new-onset hip pain or low back pain that had not been present prior to the COVID-19 pandemic and had X-ray and MRI findings suggestive of AVN of the hip, between 16 and 75 years, and provided informed consent to participate in the study.

**Exclusion Criteria**—Patients were excluded from the study if they had congenital hip pathology, metallic implants, or cardiac pacemakers. Additionally, those who were post-traumatic or post-operative, under the age of 16, did not provide consent for the study, or were lost to follow-up were also excluded.

### Study procedure

Patients underwent detailed history and clinical examinations focusing on their COVID-19 exposure and management history. Radiological investigations, including X-rays (AP view) and MRIs of the hip of all the patients, were performed to assess bony structures, joint spaces, and soft tissues. AVN was classified using the modified Ficat and Arlet classification system.^1^

Enrolled patients were then studied for factors related to AVN of the hip, with data collected on demographics, clinical findings, and radiological results. The study compared AVN cases with and without a history of COVID-19 to assess any potential association between COVID-19 and the risk of AVN.

### Data analysis

Data were analyzed using IBM SPSS version 22. Calculations were made using descriptive statistics, such as mean and standard deviation for continuous data and frequencies and percentages for categorical variables. For analysis, statistical tests such as the chi-square and t-test were employed. A *p* value of less than 0.05 was deemed statistically significant.

### Ethical clearance

The procedures followed were in accordance with the ethical standards of the responsible committee and with the Helsinki Declaration of 1975, as revised in 2000.Ethical clearance was obtained from the institutional ethics committee prior to the commencement of the study. (32278/MC/IEC/2022).

## RESULTS

### Demographic characteristics

Most participants were between the 30 and 45 years’ age group (44.2%), followed by the 15–30 years age group (30.2%). Only a small percentage (2.3%) of participants were in the 60–75 years’ age group.

The study participants are predominantly male (83.7%), with females constituting only 16.3% of the participants. The majority (74.4%) of the participants were non-COVID patients, while 25.6%were post-COVID patients. Table 1 represents the demographic distribution of our study.

### Pathological characteristics

Bilateral AVN was observed in 62.8% of the cases, with similar distributions in both non-COVID (62.5%) and post-COVID (63.6%) groups. Left-sided AVN was more seen in post-COVID patients (22.7%) compared to non-COVID patients (18.8%), while right-sided AVN was seen in non-COVID patients (18.8%) compared to post-COVID patients (13.6%). Table 2 shows the comparative distribution of sides with *p* value.

This table compares the occurrence of hip and lower back pain in patients with and without COVID. Bilateral hip pain is more frequent in COVID-positive patients (54.5%) than in COVID-negative ones (43.8%), though the difference is not statistically significant (*p* = 0.193). Left hip pain and lower back pain show almost identical frequencies between both groups, with p-values of 0.952 and 0.779, respectively, indicating no significant difference. Right hip pain is more common in COVID-negative patients (21.9%) compared to COVID-positive patients (9.1%), but this difference also lacks statistical significance (*p* = 0.183). Overall, none of the presenting complaints show a statistically significant association with COVID status, as all p-values are greater than 0.05. Tabular distribution with *p* value is represented in Table 3.

Table 4 presents the association between COVID-19 status and various comorbidities and habits among 86 patients. The data indicates a significant association (*p* value = 0.014), suggesting that the presence of comorbidities and certain habits significantly correlates with COVID-19 status. Hypertension (*p* value – 0.04) and type 2 diabetes mellitus (*p* value – 0.02) showed a statistical significant association with COVID-19.

Table 5 shows the association between COVID-19 status and steroid therapy history among 86 patients diagnosed with AVN. Among patients without a history of COVID-19 infection, 46 did not have steroid therapy ever, while 18 took steroid therapy overall. The mean dose of prednisolone was 51.74 mg + 8.8 mg/day for a median of 5 + 1.8 days. In contrast, among patients with a history of COVID-19 infection, 14 took steroid therapy, while 8 did not take steroid therapy. The *p* value of 0.002 indicates a significant association, suggesting that a history of steroid therapy is significantly correlated with COVID-19 status in patients with AVN and as an independent occurrence of AVN in patients with a history of COVID-19 with or without steroid usage. The odds ratio for AVN patients with COVID-19 therapy and steroid therapy was 4.47, suggesting a strong association of steroid therapy in COVID-19 patients with AVN.

To reduce the impact of uncontrolled confounders, we have divided them into three groups. Group A should include patients with AVN associated with COVID-19 infection, regardless of any steroid exposure. This group is essential for exploring the potential direct effects of COVID-19 on the development of AVN, separate from the influence of steroid use. Group B should encompass individuals with AVN who are not linked to COVID-19 but have been treated with steroids. This cohort is invaluable for understanding how steroid therapy impacts the incidence of AVN in the absence of a viral infection. Group C can act as a control group, consisting of patients with AVN unrelated to both COVID-19 infection and steroid therapy (Table 6).

## DISCUSSION

This study aims to explore the demographic, pathological, and treatment-related characteristics of patients with AVN of the head of the femur, emphasizing its prevalence in patients of post-COVID-19 and who underwent steroid therapy. Our study has a predominance of young males (83.7%) who are mostly in the 30–45 years’ age group (44.2%). This demographic distribution aligns with recent studies that noted a higher prevalence of AVN among males and in individuals in the age group of 20–40 which are attributed to factors such as trauma and higher exposure to risk factors like trauma and genetic predisposition.^20^ The low percentage of older participants is due to the progressive nature of AVN, which typically becomes symptomatic earlier in life, and hence, more younger patients present with symptoms.^21^

Our observation of bilateral AVN in 62.8% of cases is in line with recent studies that indicate a prevalence of bilateral AVN of up to 72%.^10,22^ Hip pain was the most common presenting complaint, apart from pain radiating to the groin and thigh, even up to the knee.^23^ Back pain is also seen in some patients which could be easily misinterpreted.^24^

Our study revealed a significant association between COVID-19 status and comorbidities and habits (*p* = 0.014). Patients with hypertension and type 2 diabetes mellitus conditions had a higher prevalence of COVID-19 (3 out of 4 for both), indicated by the low *p* value (0.020). Post-COVID patients had higher rates of hypertension (13.6% vs. 1.6%) and type 2 diabetes mellitus (13.6% vs. 1.6%) compared to non-COVID patients. A significant number of patients without comorbidities also had COVID-19 (12 out of 66), with a *p* value of 0.004. This suggests that even in the absence of comorbidities, COVID-19 was prevalent among AVN patients. The association of comorbidities with the incidence and severity of COVID-19 has been found in other studies as well.^25^ Conversely, smoking and alcohol use were less prevalent among post-COVID patients (13.6% vs. 9.4% and 0% vs. 1.6%, respectively). Research indicates that smoking was less prevalent among post-COVID patients, with a higher prevalence of smoking observed in patients with severe COVID-19 outcomes compared to non-severe outcomes.^26^ Research by Metz et al. concluded that alcohol use among post-COVID patients has been reported to decrease following the onset of the COVID-19 pandemic, although this decrease was not uniform across all patient subgroups.^27^ These observed associations of comorbidities and COVID-19 underscores the importance of managing chronic conditions and lifestyle factors in patients to improve outcomes.

A significant portion of patients with AVN (25.6%) had a history of COVID-19. 36.4% of COVID patients developed AVN despite not taking steroid therapy; hence, it shows that COVID-19 can independently be a risk factor for AVN.

This study showed a significant association between steroid therapy and COVID-19 status (*p* = 0.002). Since steroid use is the most common cause of non-traumatic AVN, bone AVN resulting from steroid treatment existed even prior to the COVID-19 epidemic. Without other risk factors, the incidence is estimated to be 21%, and with chronic or high-dose therapy, the risk is increased.^28^ Steroid therapy’s anti-inflammatory effects could interfere with the natural bone maintenance and remodeling process, which can reduce bone density if used for an extended period and at greater dosages, thus exacerbating AVN.^29^ Many other studies, like Jyothiprasanth et al., reported that the majority of AVN patients in his study with COVID-19 did not receive steroid therapy, which indicates COVID infection as a potential risk factor for AVN.^30^ Therefore, it is difficult to establish a direct association of COVID-19 or steroid therapy either solely or combined as a causative factor for AVN. This study aims to spread awareness among healthcare workers regarding AVN as a severe complication of COVID-19 and steroid therapy and highlights the necessity to exercise caution while monitoring and managing post-acute problems in COVID-19 survivors. Further research is essential to explore these relationships in greater depth and develop effective prevention and treatment strategies.

### Limitations

A small sample size of 86 individuals could hamper the establishment of a direct relationship between AVN and COVID-19, decrease the power of the study, and increase random errors.

## CONCLUSION

In individuals with AVN of the femoral head, our study demonstrates an important relationship between COVID-19, steroid use, and comorbidities such as diabetes and hypertension. Steroid therapy, which is frequently used to treat COVID-19, may increase the risk of AVN of the neck of the femur (AVN). Efficient management and mitigation of hazards are crucial for AVN patients undergoing steroid medication, especially those with comorbidities or COVID-19.

### Limitation

A larger case series with a control group could determine the direct association and risk of COVID-19 alone in the causality of AVN. A history of duration and dose of steroid therapy was not available, and this can help to identify steroid therapy dosing as a potential risk factor. The lack of a control group and small sample size limits us from doing subgroup analysis, and further research is needed in that direction.

## Acknowledgments

None.

## Conflict of interest

None.

## Authors’ contribution

SS: concept, design, definition of intellectual content, data acquisition; AB: concept, design, definition of intellectual content, data acquisition; RK: concept, design, definition of intellectual content, data acquisition; SU: concept, literature search, manuscript preparation; JT: data analysis, statistical analysis, manuscript preparation and editing; SM: literature search, manuscript preparation; AB: statistical analysis, literature search, manuscript preparation; KS: manuscript review and editing; PD: manuscript review and editing; KD: manuscript review and editing; KS: manuscript preparation, editing and review.

## Declaration statement

The manuscript has been read and approved by all authors. The contributions of each author have been specified. Each author believes that this manuscript represents honest work.

## Figures and Tables

**Table 1.** Age group distribution of the participants.

**Age group**	**Frequency**	**Percent (%)**
15–30	26	30.2
30–45	38	44.2
45–60	20	23.3
60–75	2	2.3
Total	86	100.0

**Table 2.** Side of AVN among study participants.

**Side of AVN**	**COVID Status**		***p* value**
	**No (64)**	**Yes (22)**	
Bilateral	40 (62.5%)	14 (63.6%)	0.924
Left	12 (18.8%)	5 (22.7%)	0.163
Right	12 (18.8%)	3 (13.6%)	0.640
Total	64 (100.0%)	22 (100.0%)	

**Table 3.** Presenting complaints among study participants.

**Presenting complaint**	**COVID status**		***p* value**
	**No (64)**	**Yes (22)**	
Bilateral hip pain	28 (43.8%)	12 (54.5%)	0.193
Left hip pain	12 (18.8%)	4 (18.2%)	0.952
Lower back pain	10 (15.6%)	4 (18.2%)	0.779
Right hip pain	14 (21.9%)	2 (9.1%)	0.183
Total	64 (100.0%)	22 (100.0%)	

**Table 4.** Association of COVID status versus comorbidities and habits (*p* value = 0.014) (*p* value < 0.05 = significant).

**Comorbidities and habits**	**COVID status**		***p* value**	**Total number**
	**No**	**Yes**		
Alcoholic	1	0		1
Hypertension	1	3	0.020	4
Hypertension, type 2 diabetes mellitus	1	0		1
NIL	54	12	0.004	66
Smoker	6	3	0.573	9
Smoker, type 2 diabetes mellitus	0	1		1
Type 2 diabetes mellitus	1	3	0.020	4
Total	64	22	0.014	86

**Table 5.** Association of COVID-19 status with steroid therapy history.

**Steroid therapy history**	**COVID-19 status**		***p* value**
	**No**	**Yes**	
No	46 (71.9%)	8 (36.4%)	0.002
Yes	18 (28.1%)	14 (63.6%)	0.002
Total	64	22	

**Table 6.** Subgroup analyses of the participants into three groups.

**Sr. No.**	**Variable**		**Group A**	**Group B**	**Group C**	**Sig.**
1	Age	15–30	5 (22.7)	6 (30.0)	14 (31.8)	0.065
		30–45	8 (36.4)	8 (40)	22 (50)	
		45–60	7 (31.8)	6 (30)	8 (18.2)	
		60–75	2 (9.1)			
2	Side of AVN	B/L	14 (63.6)	8 (40)	32 (72.7)	0.129
		L	5 (22.7)	7 (35)	5 (11.4)	
		R	3 (13.6)	5 (25)	7 (15.9)	
3	Presenting complaints	Bilateral	9 (40.9)	6 (30.0)	25 (56.8)	0.332
		Left	4 (18.2)	7 (35.0)	5 (11.4)	
		Lower	4 (18.2)	2 (10.0)	8 (18.2)	
		Right	5 (22.7)	5 (25.0)	6 (13.6)	
4	Steroid therapy	Yes	8 (36.4)	20 (100)	0	<0.001
		No	14 (63.6)	0	44 (100)	

Group A: patients with avascular necrosis associated with COVID-19 infection, regardless of any steroid exposure; Group B: individuals with avascular necrosis who are not linked to COVID-19 but have been treated with steroids; Group C: patients with avascular necrosis unrelated to both COVID-19 infection and steroid therapy.
